# Beyond the First Year: Epidemiology and Management of Late-Onset Opportunistic Infections After Kidney Transplantation

**DOI:** 10.3389/ti.2024.12065

**Published:** 2024-02-26

**Authors:** V. Esnault, L. Hoisnard, B. Peiffer, V. Fihman, S. Fourati, C. Angebault, C. Champy, S. Gallien, P. Attias, A. Morel, P. Grimbert, G. Melica, M. Matignon

**Affiliations:** ^1^ Assistance Publique-Hôpitaux de Paris (AP-HP), Service de Maladies Infectieuses et d’Immunologie Clinique, Centre Hospitalo-Universitaire (CHU) Henri Mondor, Créteil, France; ^2^ Fédération Hospitalo-Universitaire TRUE InnovaTive theRapy for immUne disordErs, AP-HP, Henri Mondor Hospital, Créteil, France; ^3^ INSERM, Centre d’Investigation Clinique 1430, Créteil, France; ^4^ EpiDermE Epidemiology in Dermatology and Evaluation of Therapeutics, EA7379, Paris Est Créteil University UPEC, Créteil, France; ^5^ AP-HP, Département Médico-Universitaire Médecine, CHU Henri Mondor, Créteil, France; ^6^ AP-HP, Service de Microbiologie, Département de Prévention, Diagnostic et Traitement des Infections, CHU Henri Mondor, Créteil, France; ^7^ EA DYNAMiC 7380, Faculté de Santé, University Paris-Est Créteil (UPEC), Ecole Nationale Vétérinaire d’Alfort (ENVA), USC Anses, Créteil, France; ^8^ AP-HP, Service d’Urologie, CHU Henri Mondor, Créteil, France; ^9^AP-HP, Service de Néphrologie et de Transplantation Rénale, Fédération Hospitalo-Universitaire « Innovative Therapy for Immune Disorders », CHU Henri Mondor, Créteil, France; ^10^ University of Paris-Est-Créteil, Institut National de la Santé et de la Recherche Médicale (INSERM) U955, Team 21, Institut Mondor de Recherche Biomédicale, Créteil, France

**Keywords:** kidney transplant, herpes zoster, opportunistic infections, transplant infectious disease, pneumocystis

## Abstract

Late opportunistic infections (OI) occurring beyond the first year after kidney transplantation (KT) are poorly described and not targeted by prophylactic strategies. We performed a ten-year retrospective monocentric cohort study describing epidemiology, risk factors and impact of late OI occurring 1 year after KT. We included clinically symptomatic OI requiring treatment besides BK virus nephropathy. Control groups included early OI occurring in the first year after KT, and KT recipients without OI since KT and alive with a functional allograft at 1 year. Among 1066 KT recipients, 185 (19.4%) presented a first episode of OI 21.0 (8.0–45.0) months after KT: 120 late OI (64.9%) and 65 early OI (35.1%). Late OI were mainly viral (*N* = 83, 69.2%), mostly herpes zoster (HZ) (*N* = 36, 43.4%). Pneumocystis represented most late fungal infections (*N* = 12/25, 48%). Compared to early OI, we reported more pneumocystis (*p* = 0.002) and less invasive aspergillosis (*p* = 0.01) among late OI. Patients with late OI were significatively younger at KT (54.0 ± 13.3 vs. 60.2 ± 14.3 years, *p* = 0.05). Patient and allograft survival rates between late OI and control groups were similar. Only age was independently associated with mortality. While late OI were not associated with higher mortality or graft loss, implementing prophylactic strategies might prevent such infections.

## Graphical Abstract

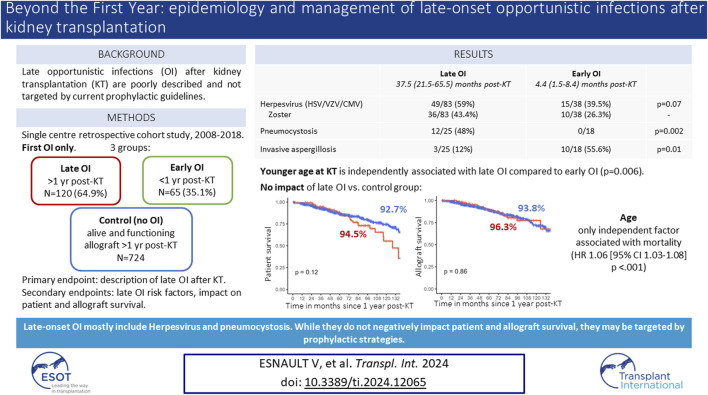

## Introduction

Kidney transplantation (KT) remains the best treatment of end-stage renal disease with better quality-of-life and longer survival than dialysis [1]. While management of kidney transplant recipients (KTR) has improved patient and allograft survival over the last decades, infections remain a major concern and represent the second cause of death within the first year post-KT [2].

Occurrence of opportunistic infections (OI) after transplantation may be considered as an inappropriate net state of immunosuppression for a given patient, resulting from a complex interaction of numerous factors among which the nature of the immunosuppressive therapy is essential [3]. OI affect up to 25% of KTR [2]. Historically, the first 6–12 months after KT represented the period most at-risk for OI, in relation with intensive immunosuppressive regimens including induction [4]. With the implementation of universal antimicrobial prophylaxis within the first year after KT, incidence of *Pneumocystis jirovecii* pneumonia (PJP) and cytomegalovirus (CMV) disease have dropped [5–7]. However, we and others have reported increasing rates of OI beyond the first 12 months after KT [8, 9], possibly related to a rising proportion of older and comorbid KTR, and identification of extended criteria donor as an independent risk factor of OI [9]. New immunosuppressive agents such as belatacept have also been associated with susceptibility to some OI including CMV disease and PJP [10, 11]. While preventive strategies are well defined within the first year after transplantation, the place for antimicrobial prophylaxis beyond 1-year post-transplantation is still lacking in current guidelines [12]. Moreover, no study comparing the epidemiology of late versus early OI is available but is mandatory to determine relevant prophylaxis.

In this context, we conducted a monocentric retrospective cohort study to describe late OI characteristics in KTR and assess the impact of such infections on patient and kidney allograft survivals.

## Materials and Methods

### Study Design and Patients

We conducted a single centre retrospective cohort study. All adult KTR engrafted between January 2008 and December 2018 in Henri Mondor Hospital (Créteil, France) were eligible apart from primary allograft non-function within 30 days after KT and combined transplantation.

All patients received infectious prophylaxis according to international guidelines [13]. CMV prophylaxis consisted in valganciclovir for intermediate (R+ treated with lymphodepleting agents) and high-risk patients (D+/R−) within 3 and 6 months after KT, respectively [7]. PJP prophylaxis consisted in trimethoprim-sulfamethoxazole (TMP-SMX) for 12 months after KT [6].

As occurrence of an OI indicates an inappropriate net state of immunosuppression [3], it may frequently lead to modification of the immunosuppressive regimen. Consequently, the first OI represents a tipping point in KTR care, and we only included the first OI for each patient. KTR with a first OI occurring beyond the first year post-transplantation were included in the late OI group (LOI). We chose the cut-off of 12 months after KT as TMP-SMX is withdrawn at this time point in our centre. Two control groups were defined: 1) KTR with a first OI occurring within the first year after KT (early OI, EOI); 2) KTR with no history of OI since transplantation and alive with a functioning allograft for at least 1 year after KT (no-OI group).

### OI Definition and Collection

In the absence of a standardized list of OI in solid organ transplant recipients, definition of OI was based on the 1993 revised classification system for OI in the setting of human immunodeficiency virus (HIV) infection [14], on international guidelines [6, 7], and the concertation of two senior Infectious Disease and Kidney Transplantation specialists. We analysed symptomatic OI requiring therapy without restriction to hospitalized OI, except for BK virus nephropathy (BKVN) for which no treatment is available. The following pathogens and infections were considered (a complete definition of OI is provided in Supplementary Data):

-Bacteria: *Nocardia* sp., *Mycobacterium tuberculosis* and non-tuberculous mycobacteria, *Listeria monocytogenes*, *Legionella pneumophila*.
-Virus: severe herpes simplex virus (HSV) infections (encephalitis, pneumonitis or other organ involvement requiring appropriate antiviral treatment); severe varicella-zoster virus (VZV) infections [encephalitis, pneumonitis, herpes zoster (HZ) requiring appropriate antiviral treatment]; hepatitis B (HBV) reactivation, hepatitis E infection (HEV); CMV disease; Human-Herpes virus 8 (HHV8)-associated Kaposi sarcoma; JC virus-associated progressive multifocal leukoencephalopathy (PML); chronic norovirus infection; disseminated and severe localized adenovirus disease; histologically proven BK virus-associated nephropathy (BKVN) (i.e., no presumptive BKVN).
-Fungi: invasive candidiasis and rare yeast disseminated infections such as *Trichosporon* spp.; *Cryptococcus neoformans*; invasive mold diseases (aspergillosis, mucormycosis, fusariosis); *Pneumocystis jirovecii* pneumonia.
-Parasites: *Toxoplasma gondii*, *Microsporidium* sp, *Cryptosporidium* sp, *Leishmania* sp.

OI were identified in our local KT database, which prospectively collects patient data from registration on KT waiting list to engraftment, as well as every significant in- and out-patient event occurring afterwards. All events are implemented by a clinical research associate specialized in KT. OI characteristics were retrospectively collected from patients’ medical records and independently reviewed and validated by two Infectious Diseases specialists.

### Outcomes

The primary endpoint was description of late OI after KT. Secondary endpoints were 1) risk factors of late OI compared to early OI after KT and 2) impact of late OI on overall survival and kidney allograft survival after KT. Allograft loss was considered if dialysis was needed or if the estimated glomerular filtration rate (eGFR) was below 15 mL/min/1.73 m^2^.

### Covariates

We collected data about KTR characteristics [age, sex, underlying nephropathy, extended criteria donor (ECD), biological data] and immunosuppressive therapy (induction and maintenance regimen, rejection therapy). ECD was defined as a donor older than 60 years or between 50 and 60 years, with two of the three following criteria: 1) hypertension; 2) pre-retrieval serum creatinine >1.50 mg/dL; and 3) cerebrovascular cause of brain death [15]. Delayed graft function was considered in case of haemodialysis within the first 7 days after KT. The eGFR was estimated using CKD-EPI formula [16]. Acute rejection episodes were histologically proven and analysed according to updated Banff classification [17]. Our local immunosuppressive protocol is provided in Supplementary Methods. Conversion to belatacept as maintenance regimen was considered for analysis if treatment had been initiated at least 1 month before the first OI episode.

### Statistical Analysis

Continuous variables were described by mean (standard deviation, SD) or median (interquartile range, IQR) as appropriate and categorical variables by number and percentage. We used t-test or Wilcoxon test for continuous variables and Chi-2 or Fisher exact tests for categorical variables. Logistic regression models were performed for multivariable analyses, which included all variables with a *p*-value ≤0.2 in univariable analysis.

In the primary analyses, we studied late OI and early OI groups. Patients were followed from the first OI episode to allograft loss, death from any cause or until 31st December 2020 (end of study period) whichever occurred first. Overall survival, allograft survival and survival without allograft loss were described with Kaplan Meier curves, using the occurrence of the first OI as baseline. Hazard ratios were estimated by Cox model regressions which included variables known to be associated with both patient and allograft survival: sex and age. Proportional-hazards assumption was formally tested by using Schoenfeld residuals. As BKVN is an independent risk factor of graft loss, we then performed a sensitivity analysis excluding patients with BKVN.

We also performed a secondary analysis comparing survival rates between late OI and no-OI groups for which baseline was set at 1-year post-transplantation, i. e., conditional survival analysis beyond 1 year.

A *p*-value <0.05 was considered significant. Tests were two-tailed. To account for multiple testing, we used Benjamini-Hochberg method as appropriate. Statistical analyses were carried out using R 3.6.2.

## Results

**FIGURE 1:**
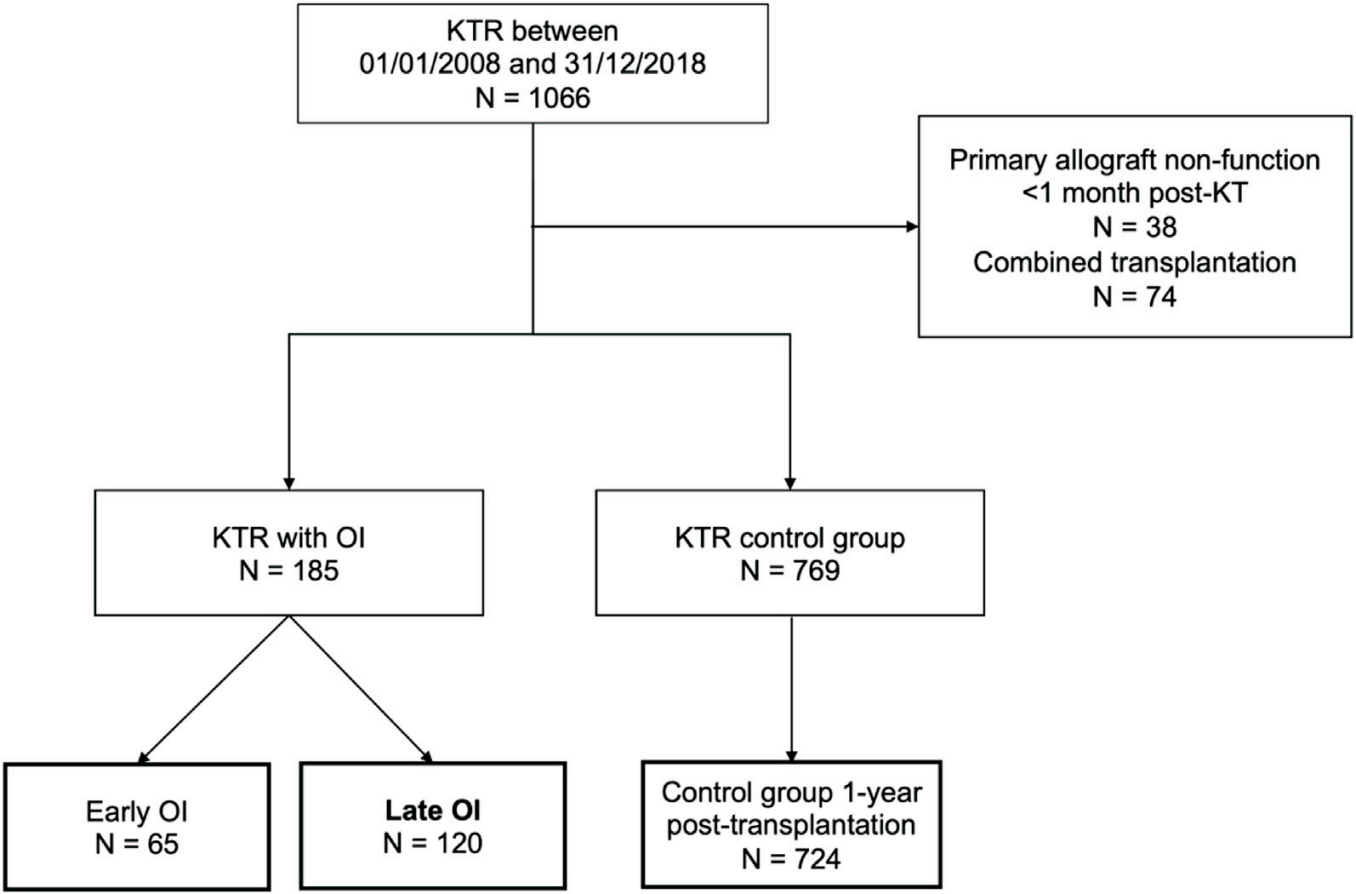
Flowchart of the patients included in the study. Opportunistic infections (OI) were divided into early OI (occurring within the first year after kidney transplantation) and late OI (occurring beyond the first year post-transplantation). The control group included 724 kidney transplant recipients (KTR) without OI who were alive with a functional kidney allograft at least 1 year after transplantation.

Between January 2008 and December 2018, 1066 KT were performed in our centre and 954 KTR were included in the study (Figure 1). Among those, 185 (19.4%) presented a first OI in a median time of 21.0 (8.0–45.0) months after KT: 120 late OI (64.9%) and 65 early OI (35.1%). The control group with no history of OI included 724 KTR alive up to 1 year after KT.

Late and early OI occurred 37.5 (21.5–65.5) and 4.4 (1.5–8.4) months after KT, respectively. Most late OI were viral (*N* = 83, 69.2%) and fungal (*N* = 25, 20.8%) infections, while bacterial and parasitic OI were scarce (*N* = 5, 4.2% and *N* = 7, 5.8%, respectively) (Table 1). Among late OI, leading viral infections were HZ (*N* = 36, 43.4%), BKVN (*N* = 15, 18.1%) and CMV disease (*N* = 11, 13.3%). Fungal infections consisted mainly in PJP (*N* = 12, 48%) and invasive candidiasis (*N* = 7, 28%). Other invasive fungal infections included invasive aspergillosis (*N* = 3, 12%), cryptococcosis (*N* = 2, 8%) and mucormycosis (*N* = 1, 4%). Compared to early OI, we reported significatively more PJP (*N* = 12, 48% vs. *N* = 0, *p* = 0.002) occurring in a median time of 51.7 (23.8–76.5) months, and significatively less invasive aspergillosis among late OI (*N* = 3, 12% vs. *N* = 10, 55.6%, *p* = 0.01, respectively). Invasive aspergillosis occurred 4.0 (1.3–8.5) months after KT among early OI.

**TABLE 1:** Description of late opportunistic infections and comparison to early opportunistic infections.

	Total *N* = 185	Late opportunistic infection *N* = 120	Early opportunistic infection *N* = 65	*p*-value
Type of infection				0.33
Viral, *N* (%)	121 (65.4)	83 (69.2)	38 (58.5)	
Fungal, *N* (%)	43 (23.2)	25 (20.8)	18 (27.7)	
Bacterial, *N* (%)	11 (5.9)	5 (4.2)	6 (9.2)	
Parasitic, *N* (%)	10 (5.4)	7 (5.8)	3 (4.6)	
Viral infections	*N* = 121	*N* = 83	*N* = 38	
Herpesviridae (CMV/HSV/VZV)	64 (52.9)	49 (59.0)	15 (39.5)	0.07
Herpes zoster	46 (38.0)	36 (43.4)	10 (26.3)	—
CMV disease	15 (12.4)	11 (13.3)	4 (10.5)	—
BK virus nephropathy	27 (22.3)	15 (18.1)	12 (31.6)	0.16
Norovirus/adenovirus	12 (9.9)	7 (8.4)	5 (13.2)	0.51
HBV/HEV	7 (5.8)	6 (7.2)	1 (2.6)	0.43
HHV8	11 (9.1)	6 (7.2)	5 (13.2)	0.32
Fungal infections	*N* = 43	*N* = 25	*N* = 18	
*Aspergillus* spp.	13 (30.2)	3 (12.0)	10 (55.6)	0.01
*Pneumocystis jirovecii* pneumonia	12 (27.9)	12 (48.0)	0 (0.0)	0.002
*Candida* spp.	11 (25.6)	7 (28.0)	4 (22.2)	0.74
Cryptococcosis	6 (14.0)	2 (8.0)	4 (22.2)	0.22
Mucormycosis	1 (2.3)	1 (4.0)	0 (0.0)	
Bacterial infections	*N* = 11	*N* = 5	*N* = 6	
*Legionella*	5 (45.5)	3 (60.0)	2 (33.3)	
*Nocardia* spp.	2 (18.2)	0 (0.0)	2 (33.3)	
Tuberculosis	3 (27.3)	1 (20.0)	2 (33.3)	
Non-tuberculous mycobacteria	1 (9.1)	1 (20.0)	0 (0.0)	
Parasitic infections	*N* = 10	*N* = 7	*N* = 3	
Microsporidiosis	2 (20.0)	2 (28.6)	0 (0.0)	
Cryptosporidiosis	5 (50.0)	3 (42.8)	2 (66.7)	
Toxoplasmosis	3 (30.0)	2 (28.6)	1 (33.3)	

CMV, cytomegalovirus; HSV, herpes simplex virus; VZV, varicella-zoster virus; HBV, hepatitis B virus; HEV, hepatitis E virus.

Characteristics of KTR from the late OI and early OI groups are presented in Table 2. KTR with a first OI occurring beyond 12 months post-allograft were significatively younger at the time of KT (54.0 ± 13.3 vs. 60.2 ± 14.3 years, *p* = 0.05). Induction and maintenance immunosuppressive regimens were similar in both groups. Occurrence of acute rejection in the year preceding the OI was significantly less reported among the late OI group (4.2% vs. 18.5%, *p* = 0.04). There was no difference between the two groups in terms of occurrence of CMV viremia prior to the OI, new-onset diabetes after transplantation or switch to belatacept (Table 2). Among PJP episodes, lymphocytopenia (defined by an absolute lymphocyte count [ALC] <1,000/mm^3^) in the year preceding the OI was reported in 6/8 (75%) KTR with available data.

**TABLE 2:** Patients characteristics at the time of kidney transplantation and during follow-up.

	Total	Late opportunistic infection *N* = 120	Early opportunistic infection *N* = 65	*p*-value
Recipient				
Age, mean ± SD	56.2 ± 14.0	54.0 ± 13.3	60.2 ± 14.3	0.05
Male sex, *N* (%)	124 (67.0)	75 (62.5)	49 (75.4)	0.34
Dialysis before KT, *N* (%)	168 (92.3)	106 (90.6)	62 (95.4)	0.70
Time on dialysis (years), median (IQR)	4.2 (2.2–6.2)	4.3 (2.2–6.6)	4.1 (2.2–6.0)	0.84
History of non-kidney SOT, N (%)	4 (2.2)	0 (0.0)	4 (6.2)	0.07
Diabetes^a^, *N* (%)	39 (26.2)	18 (19.6)	21 (36.8)	0.13
Underlying nephropathy				0.44
Glomerulopathy, *N* (%)	27 (14.8)	19 (16.2)	8 (12.3)	
Diabetes, *N* (%)	26 (14.3)	11 (9.4)	15 (23.1)	
Genetic, *N* (%)	18 (9.9)	13 (11.1)	5 (7.7)	
Autoimmune disease, *N* (%)	6 (3.3)	3 (2.6)	3 (4.6)	
Other, *N* (%)	45 (24.7)	31 (26.5)	14 (21.5)	
Unspecified, *N* (%)	60 (33.0)	40 (34.2)	20 (30.8)	
Biological characteristics				
Leukocytes (/mm^3^), median (IQR)	6200 (5200–7900)	6200 (5300–7900)	6100 (5200–7800)	0.95
Lymphocytes (/mm^3^), median (IQR)	1,300 (1,000–1,700)	1,300 (1,000–1,750)	1,200.0 (825–1,500)	0.41
Lymphocytes <1,000/mm^3^, *N* (%)	41 (24.3)	24 (22.4)	17 (27.4)	0.84
VIH, *N* (%)	6 (3.3)	5 (4.3)	1 (1.5)	0.70
VHC, *N* (%)	6 (3.4)	3 (2.6)	3 (4.7)	0.88
Donor				
Age, mean ± SD	60.0 ± 15.1	58.5 ± 15.0	62.8 ± 14.8	0.21
Living donor, *N* (%)	14 (7.7)	12 (10.3)	2 (3.1)	0.41
Extended criteria donor, *N* (%)	105 (57.7)	59 (50.4)	46 (70.8)	0.07
Kidney transplantation				
DSA, *N* (%)	34 (21.9)	23 (23.0)	11 (20.0)	0.95
CMV serostatus (D/R), *N* (%)				1
D−/R−	12 (6.6)	8 (6.8)	4 (6.2)	
D−/R+	62 (34.1)	39 (33.3)	23 (35.4)	
D+/R−	23 (12.6)	16 (13.7)	7 (10.8)	
D+/R+	85 (46.7)	54 (46.2)	31 (47.7)	
Cold ischemia time (hours) median (IQR)	15.9 (12.6–20.1)	15.2 (12.0–20.0)	16.8 (14.0–21.0)	0.07
Induction therapy				
Anti-CD25 mAbs, *N* (%)	92 (50.5)	63 (53.8)	29 (44.6)	0.62
Polyclonal antithymocyte globulin, *N* (%)	87 (47.8)	51 (43.6)	36 (55.4)	0.41
Maintenance immunosuppressive regimen				
Calcineurin inhibitors, *N* (%)				
Ciclosporin	35 (19.2)	25 (21.4)	10 (15.4)	0.70
Tacrolimus	160 (87.9)	105 (89.7)	55 (84.6)	0.70
Mycophenolate mofetil, *N* (%)	156 (85.7)	100 (85.5)	56 (86.2)	1
mTOR inhibitors, *N* (%)	25 (13.7)	15 (12.8)	10 (15.4)	0.95
Corticosteroids, *N* (%)	182 (100)	117 (100)	65 (100)	
During follow-up				
Rejection treated within the year preceding OI, *N* (%)	17 (9.2)	5 (4.2)	12 (18.5)	0.04
CMV viremia, *N* (%)	50 (27.0)	35 (29.2)	15 (23.1)	0.72
New-onset diabetes, *N* (%)	51 (27.6)	34 (28.3)	17 (26.2)	0.99
Switch to belatacept, *N* (%)	21 (11.4)	14 (11.7)	7 (10.8)	1

IQR, interquartile range; KTR, kidney-transplant recipient; CMV, cytomegalovirus; DSA, donor specific antibodies; mAbs, monoclonal antibodies; mTOR, mammalian target of rapamycin; SD, standard deviation; SOT, solid organ transplant.

^a^ Significant missing data (36/185, 19.5%).

In multivariable analysis, younger age at KT (*p* = 0.006) and less acute rejection within the year before OI (*p* = 0.003) were significantly associated with late OI compared to early OI (Table 3).

**TABLE 3:** Risk factors of late opportunistic infections compared to early opportunistic infections.

	Univariable analysis		Multivariable analysis	
	OR [95% CI]	*p*-value	ORa [95% CI]	*p*-value
Age	0.97 [0.94–0.99]	0.005	0.97 [0.94–0.99]	0.006
Male sex	0.54 [0.27–1.05]	0.08	0.64 [0.31–1.29]	0.22
Rejection treated within the year preceding OI, *N* (%)	0.19 [0.06–0.55]	0.003	0.18 [0.05–0.52]	0.003

OI, opportunistic infection; CMV, cytomegalovirus.

**FIGURE 2:**
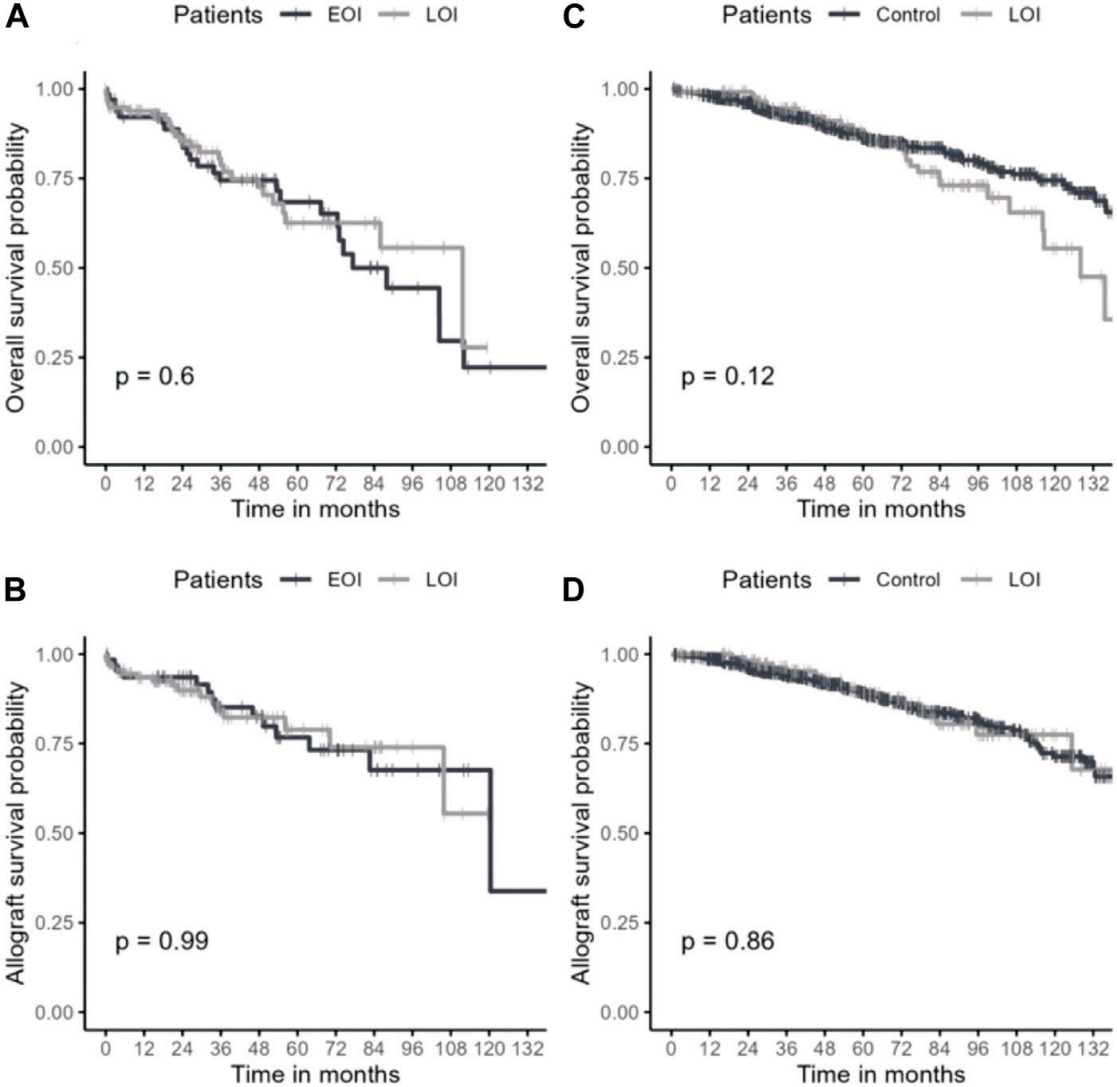
Overall survival and kidney allograft survival in late OI and control groups. Overall survival **(A)** and kidney allograft survival **(B)** after OI episode. Overall survival **(C)** and kidney allograft survival **(D)** after 1 year of transplantation. OI: opportunistic infection; Early/Late OI: Opportunistic infections occurring within the first-year post-transplantation (EOI) and after the first-year post-transplantation (LOI); Control: kidney transplant recipient without opportunistic infection alive with a functional allograft at least 1 year after transplantation.

Mean follow-up was of 68.7 (±37.1) months. Overall and allograft survivals in late and early OI groups are presented in Figures 2A, B. Thirty-six months after OI, overall survival rates were 78.7% in the late OI group and 74.5% in the early OI group (*p* = 0.6). OI-related mortality occurred in 5/60 (8.33%) patients: 2/27 (7.4%) in the early OI group (one *Aspergillus fumigatus* endocarditis and one Kaposi sarcoma), and 3/33 (9.0%) patients in the late OI group (two PJP and one CMV disease). Thirty-six months after OI, allograft survival rates in the late OI and early OI groups were similar (84.3% and 85.2%, respectively, *p* = 0.99). As BKVN may influence kidney allograft survival, we performed kidney allograft survival analysis excluding those OI; results were similar (85.1% and 88.2%, respectively, *p* = 0.70). Compared to early OI, occurrence of late OI was not associated with mortality (adjusted HR 1.22 [95% CI 0.69–2.17], *p* = 0.49). Age was an independent factor of mortality (aHR 1.06 [95% CI 1.03–1.08], *p* < 0.001). Gender was not associated with mortality (male sex aHR 1.24 [95% CI 0.67–2.30], *p* = 0.50).

To specifically assess the impact of late OI on overall and allograft survivals, we compared late OI to the no-OI control group. Characteristics from both groups were similar (Supplementary Table S1). We reported no difference in conditional survival rates at 36 months between the two groups (94.5% vs. 92.7%, respectively, *p* = 0.12) (Figures 2C, D). Compared to the control group, late OI were not associated with mortality (aHR 1.16 [95% CI 0.76–1.78], *p* = 0.49) while age was (aHR 1.08 [95% CI 1.07–1.10], *p* < 0.001). Kidney allograft survival was similar in both groups (LOI 96.3% vs. no-OI 93.8%, *p* = 0.86).

Finally, we specifically analysed the 33 (17.8%) KTR with OI converted from calcineurin inhibitors to belatacept. Among those, 21 (63.6%) presented a first episode of OI after initiation of belatacept: 14 (66.6%) late OI and 7 (33.3%) early OI (Supplementary Table S2). Time between conversion and occurrence of OI was 10.7 (2.6–22.0) months: 17.1 (11.2–24.8) months for LOI and 2.6 (2.3–2.7) months for EOI. Among converted patients with late OI, viral infections were predominant (*N* = 9, 64.3%), mostly HZ and CMV disease, followed by fungal infections (*N* = 4, 28.6%), all PJP.

## Discussion

In this large retrospective monocentric study spanning across 10 years of KTR follow-up, we reported for the first time the description of late OI occurring beyond 1 year after transplantation. PJP and HZ were the most frequently identified late OI. Occurrence of a first OI beyond 12 months post-KT was associated with younger age at transplantation. Conversely, we showed that early OI episodes were more frequent in older KTR and associated with rejection. Late-onset OI had no deleterious impact on either patient or allograft survivals.

We only analysed the first OI for each patient, considering that the first occurrence of an OI represents a tipping point in KTR management that require immunosuppression adaptations because of an inappropriate net state of immunosuppression [3]. Those modifications may lead to *de novo* donor-specific antibodies (DSA) development, antibody-mediated rejection with negative impact on kidney allograft survival [18]. Here, we reported an incidence of OI of 19% consistent with the literature (10%–25%) [8, 19]. We consider that our database captured all OI after KT, including episodes treated outside the hospital system for which the transplantation centre was systematically reached to discuss specific treatment and immunosuppression management. While our results highlight the efficacy of codified infectious prophylaxis in reducing the burden of early OI after transplantation, late-onset OI were actually predominant in our study and are currently not targeted by preventive strategies [12]. We confirmed previous data suggesting two incidence peaks of OI after KT, one within 6 months and one up to 3 years post-allograft [8, 19].

The majority of late OI were viral infections, mainly HZ. We and others had already emphasized HZ predominance after KT, which occurs in up to 10% of KTR, mostly beyond the second year post-transplantation [9, 20]. Age >50 years and steroids have been identified as risk factors, while CMV prophylaxis might be protective [20]. HZ complications are described in almost a quarter of KTR, especially in those >50 years old, and include postherpetic neuralgia, disseminated disease and cranial nerve involvement [20]. In older adults, zoster vaccine markedly reduces HZ incidence and morbidity as well as postherpetic neuralgia, regardless of VZV serology status [21–23]. In France, zoster vaccination using a live attenuated vaccine is currently recommended in all adults older than 65 years [24]. The future availability of the inactivated zoster vaccine in France should enable vaccination after KT as well [25]. Until then, we suggest that live attenuated zoster vaccination should be implemented systematically among patients awaiting KT, regardless of age.

In our study, the second predominant late OI was PJP, which occurred over 4 years after KT. No PJP was described during the first year post-transplantation, as expected with universal TMP-SMX prophylaxis prescribed for 12 months in our centre [6, 25]. Previous studies focusing on late PJP described a high burden of disease between 1 and 2 years post-transplantation occurring after prophylaxis discontinuation, with identification of an ALC <1,000/mm^3^ in the year prior to PJP as a risk factor [12, 26, 27]. Consecutively, resuming TMP-SMX prophylaxis in case of ALC <1,000/mm^3^ or maintaining life-long TMP-SMX should be discussed and evaluated in further studies [12].

Additionally, we reported a rare but significant predominance of invasive aspergillosis during the first-year post-transplantation. Incidence rate of invasive aspergillosis after KT is around 0.5%–4% and most episodes occur early, with negative impact on patient and allograft survival [28–32]. Whether the infection develops as a consequence of pre-transplantation colonisation or not remains unclear. Risk factors of invasive aspergillosis after KT include high and prolonged duration of corticosteroids, dialysis requirement after transplantation and duration of pretransplant haemodialysis [30, 32–34]. Currently, no prophylactic strategy is recommended before kidney transplantation or in the following year [35]. While there is no place for antifungal prophylaxis in this population, we suggest that non-invasive preventive strategies, such as systematic pre-transplantation sinus CT-scan to detect pauci- or asymptomatic *Aspergillus* sinusitis, should be evaluated in prospective studies. Indeed, non-invasive fungal sinusitis represents a significant risk factor of invasive fungal infections in immunocompromised individuals [36].

We also found an association between older age at transplantation and early OI. Aged KTR experience increased rates of infection probably due to immune senescence [37]. Several specific mechanisms have been described, such as accelerated aging of the CD8^+^ T cell after CMV infection and immune senescence of innate T cells [38]. Chronic kidney disease can also accelerate immune aging [39]. Our result may be the consequence of immune system exhaustion combined with the required immunosuppression. History of rejection in the year preceding the OI was also significantly more frequent in the early OI group, reflecting that increasing immunosuppression in the early period is a potent risk factor for OI.

The subgroup analysis of KTR switched from CNI to belatacept was performed *a priori* considering the higher risk of OI recently described in those KTR [10, 11]. As proportion of belatacept-switched KTR was similar in both OI groups, our data suggested that belatacept was not a risk factor of late OI. However, the small sample size prevented any definitive conclusion.

We recognize our study’s limits, the first one being its monocentric retrospective design. However, this ensured homogeneous immunosuppressive regimens—even if no CNI level information was available—rejection management and infectious prophylaxis over a ten-year period. Despite the lack of a standardized classification of OI in non-HIV immunocompromised patients, we included OI based on their clinical and therapeutic impact and validated this selection by a consensus of experienced specialists, in the light of current literature. For instance, we only included CMV disease (as defined by the evidence of CMV infection with attributable symptoms [7]), for which treatment is mandatory; we did not consider isolated CMV DNAemia for which neither therapy nor systematic surveillance are recommended after KT [7]. Analysing the first OI only for each KTR prevented us from identifying risk factors of recurrent OI. Finally, future research should focus on all severe community infections, considering that bacterial infections represent the most common cause of infection-related mortality and appropriate prophylactic strategies are still limited.

## Conclusion

Late-onset OI are currently predominant after kidney transplantation among young KTR and mostly include HZ and PJP. While we did not report a negative impact of late OI on patient and allograft survival, preventive strategies should be discussed and evaluated in prospective cohort studies.

## Data Availability

The raw data supporting the conclusions of this article will be made available by the authors, without undue reservation.

## Abbreviations

ALC, absolute lymphocyte count; CMV, cytomegalovirus; ECD, extended criteria donor; eGFR, estimated glomerular filtration rate; EOI, early opportunistic infection; HZ, herpes zoster; KT, kidney transplantation; KTR, kidney transplantation recipient; LOI, late opportunistic infection; PJP, *Pneumocystis jirovecii* pneumonia; VZV, varicella zoster virus.
