# Supplementary material for: Beyond the First Year: Epidemiology and Management of Late-Onset Opportunistic Infections After Kidney Transplantation
Source: Transpl Int. 2024 Feb 26;37:12065. doi: 10.3389/ti.2024.12065 (PMC10926380; doi:10.3389/ti.2024.12065)
Supplement: Supplementary file 1 [file DataSheet1.docx]

**Supplementary Data**

**Supplementary Methods**

**OI definition**

Infections caused by uncommon pathogens or by common pathogens with unusual and more severe forms, especially if modification of the immunosuppressive treatment is required for management of the infection.

The following pathogens, diseases or syndromes were included:

- Bacteria: *Listeria* *monocytogenes* (central nervous system [CNS] involvement); *Nocardia* sp. (CNS, lung, extensive cutaneous and/or disseminated disease ≥2 sites); *Legionella pneumophila* pneumonia; *Mycobacterium tuberculosis* (CNS, extra-pulmonary and extensive pulmonary involvement) and non-tuberculous mycobacteria (diagnosis criteria according to international guidelines (1)).

- Virus:

- severe herpes simplex virus (HSV) infections including encephalitis, pneumonitis or other organ involvement requiring appropriate antiviral treatment and tapering of immunosuppression;
- varicella-zoster virus (VZV) infections including encephalitis, pneumonitis, zoster requiring appropriate antiviral treatment and tapering of immunosuppression);
- hepatitis B (HBV) reactivation, hepatitis E (HEV);
- CMV disease: evidence of CMV infection with attributable symptoms, including CMV syndrome (defined by the association of fever, malaise, leukopenia, and/or thrombocytopenia), and tissue invasive CMV (“end organ”) disease (including pneumonia [CMV-DNA in bronchoalveolar lavage], central nervous system disease [CMV in cerebrospinal fluid], colitis, retinitis [confirmed by an ophthalmologist] or cholangitis) (2)**;**
- Human-Herpes virus 8 (HHV8) associated Kaposi sarcoma;
- JC virus-associated progressive multifocal leukoencephalopathy (PML);
- Histologically proven BK virus-associated nephropathy (BKVN). In our center, a kidney allograft biopsy with SV40 immunostaining is systematically performed in case of BK viremia >3 log copies/ml.
- Chronic norovirus infection defined as the persistence of symptoms for ≥4 weeks or the necessity of rehospitalization because of norovirus-ascribed diarrhea, in combination with positive PCR results in at least one subsequent stool sample (3); disseminated and severe localized adenovirus disease (4);

- Fungi:

- Proven or probable invasive candidiasis including candidemia (*Candida* spp isolated from blood cultures) and deep-seated candidiasis (intra-abdominal candidiasis (IAC), osteomyelitis, septic arthritis, mediastinitis, endophthalmitis, endocarditis, upper urinary tract infections) with or without candidemia (5,6); severe oropharyngeal candidiasis and esophagitis;
- rare yeast such as *Trichosporon* spp (7);
- *Cryptococcus* *neoformans* (including meningitis, fungemia, disseminated infection involving ≥2 sites) (7);
- proven or probable invasive mold diseases (*Aspergillus* sp, Mucorales, *Fusarium* sp.) (7);
- invasive infections caused by dimorphic fungi such as *Histoplasma capsulatum* (7);
- *Pneumocystis jirovecii* pneumonia.

- Parasites: *Toxoplasma gondii* (central nervous system involvement); *Microsporidium* sp, *Cryptosporidium* sp associated with chronic diarrhea (>1 month); visceral leishmania.

**Immunosuppressive Regimen:**

Kidney allograft recipients with no anti-HLA donor-specific antibodies (DSA) before transplantation or calculated panel reactive antibodies lower than 50%, received basiliximab induction and calcineurin inhibitors (CNI), mycophenolate mofetil (MMF) and steroids maintenance up to December 2016 and CNI, mTOR inhibitors (mTORi) and steroids maintenance from December 2016 to December 2018. Target troughs of tacrolimus and cyclosporine were 7-8 ng/mL and 150-175 ng/mL respectively until 12 months after transplantation, and 5 ng/mL and 100-125 ng/mL respectively after 12 months when associated with MMF. In case of association with mTORi, target troughs of tacrolimus and cyclosporine were 5-6 ng/mL and 100-125 ng/mL, respectively. MMF dose was 2000 mg per day within the first year and 1000 mg per day afterwards. Steroid dose was 20 mg per day for two weeks then tapered progressively to 5 mg per day two months after transplantation.

Regarding kidney allograft recipients with anti-HLA DSA before transplantation or calculated panel reactive antibodies higher than 50%, induction consisted in thymoglobulin and maintenance included CNI, MMF and steroids. Target troughs of tacrolimus and cyclosporine were 7-8 ng/mL and 150-175 ng/mL respectively until 12 months after transplantation, and 5 ng/mL and 100-125 ng/mL respectively after 12 months. MMF dose was 2000 mg per day within the first year and 1000 mg per day afterwards. Steroid dose was 20 mg per day for two weeks then tapered progressively to 5 mg per day two months after transplantation.

**References**

1. Griffith DE, Aksamit T, Brown-Elliott BA, Catanzaro A, Daley C, Gordin F, et al. An Official ATS/IDSA Statement: Diagnosis, Treatment, and Prevention of Nontuberculous Mycobacterial Diseases. Am J Respir Crit Care Med. 15 févr 2007;175(4):367‑416.

2. Kotton CN, Kumar D, Caliendo AM, Huprikar S, Chou S, Danziger-Isakov L, et al. The Third International Consensus Guidelines on the Management of Cytomegalovirus in Solid-organ Transplantation. Transplantation. juin 2018;102(6):900‑31.

3. Angarone M, Snydman DR, AST ID Community of Practice. Diagnosis and management of diarrhea in solid-organ transplant recipients: Guidelines from the American Society of Transplantation Infectious Diseases Community of Practice. Clin Transplant. sept 2019;33(9):e13550.

4. Al-Heeti OM, Cathro HP, Ison MG. Adenovirus Infection and Transplantation. Transplantation. 1 mai 2022;106(5):920‑7.

5. Bassetti M, Azoulay E, Kullberg BJ, Ruhnke M, Shoham S, Vazquez J, et al. EORTC/MSGERC Definitions of Invasive Fungal Diseases: Summary of Activities of the Intensive Care Unit Working Group. Clin Infect Dis Off Publ Infect Dis Soc Am. 12 mars 2021;72(Suppl 2):S121‑7.

6. Pappas PG, Lionakis MS, Arendrup MC, Ostrosky-Zeichner L, Kullberg BJ. Invasive candidiasis. Nat Rev Dis Primer. 11 mai 2018;4(1):1‑20.

7. De Pauw B, Walsh TJ, Donnelly JP, Stevens DA, Edwards JE, Calandra T, et al. Revised definitions of invasive fungal disease from the European Organization for Research and Treatment of Cancer/Invasive Fungal Infections Cooperative Group and the National Institute of Allergy and Infectious Diseases Mycoses Study Group (EORTC/MSG) Consensus Group. Clin Infect Dis Off Publ Infect Dis Soc Am. 15 juin 2008;46(12):1813‑21.

**Supplementary Results**

**Supplementary Table S1:** Clinical and biological characteristics of patients with late opportunistic infection and control group without opportunistic infection at the time of kidney transplantation

|  | Total | | Late opportunistic infection  N=120 | No opportunistic infection  N=724 | p-value |
| --- | --- | --- | --- | --- | --- |
| *Recipient* | | |  |  |  |
| Age, mean ± SD | 52.0 ± 13.9 | | 54.0 ± 13.3 | 51.7 ± 14.0 | 0.41 |
| Male sex, N (%) | 521 (61.7) | | 75 (62.5) | 446 (61.6) | 1 |
| Dialysis before KT, N (%) | 727 (87.1) | | 106 (90.6) | 621 (86.5) | 0.72 |
| Time on dialysis (years), median (IQR) | 3.7 (2.1-5.9) | | 4.3 (2.2-6.6) | 3.6 (2.1-5.9) | 0.72 |
| History of non-kidney SOT, N (%) | 8 (1.0) | | 0 (0.0) | 8 (1.1) | 0.95 |
| Diabetes* | 142 (21.6) | | 18 (19.6) | 124 (21.9) | 1 |
| *Underlying nephropathy* | |  |  |  |  |
| Glomerulopathy, N (%) | 139 (16.6) | | 19 (16.2) | 120 (16.7) |  |
| Diabetes, N (%) | 96 (11.5) | | 11 (9.4) | 85 (11.8) |  |
| Genetic, N (%) | 99 (11.9) | | 13 (11.1) | 86 (12.0) |  |
| Autoimmune disease, N (%) | 29 (3.5) | | 3 (2.6) | 26 (3.6) |  |
| Other, N (%) | 194 (23.2) | | 31 (26.5) | 163 (22.7) |  |
| Unspecified, N (%) | 278 (33.3) | | 40 (34.2) | 238 (33.1) |  |
| *Biological characteristics* | |  |  |  |  |
| Leucocytes (/mm^3^), median (IQR) | 6.4 (5.2-7.9) | | 6.2 (5.3-7.9) | 6.4 (5.2-7.9) | 1 |
| Lymphocytes (/mm^3^), median IQR | 1300.0 (1000.0- 1700.0) | | 1300.0 (1000.0-1750.0) | 1300.0 (1000.0-1700.0) | 1 |
| Lymphocytes <1000/mm^3^, N (%) | 164 (22.3) | | 24 (22.4) | 140 (22.3) | 1 |
| HIV, N (%) | 37 (4.4) | | 5 (4.3) | 32 (4.5) | 1 |
| HCV, N (%) | 28 (3.5) | | 3 (2.6) | 25 (3.6) | 1 |
| *Donor* |  | |  |  |  |
| Age, mean ± SD | 55.6 ± 15.7 | | 58.5 ± 15.0 | 55.1 ± 15.7 | 0.17 |
| Living donor, N (%) | 114 (13.7) | | 12 (10.3) | 102 (14.2) | 0.72 |
| Extended criteria donor, N (%) | 383 (46.0) | | 59 (50.4) | 324 (45.3) | 0.72 |
| *Kidney transplantation* | | |  |  |  |
| DSA, N (%) | 179 (25.8) | | 23 (23.0) | 156 (26.3) | 0.95 |
| CMV serostatus (D/R) |  | |  |  | 0.72 |
| D-/R- | 77 (9.2) | | 8 (6.8) | 69 (9.6) |  |
| D-/R+ | 273 (32.8) | | 39 (33.3) | 234 (32.7) |  |
| D+/R- | 79 (9.5) | | 16 (13.7) | 63 (8.8) |  |
| D+/R+ | 404 (48.5) | | 54 (46.2) | 350 (48.9) |  |
| Cold ischemia time (hours) median (IQR) | 16.0 (12.2-20.1) | | 15.2 (12.0-20.0) | 16.2 (12.3- 20.2) | 0.72 |
| *Induction therapy* |  | |  |  |  |
| Anti-CD25 mAbs, N (%) | 348 (41.7) | | 63 (53.8) | 285 (39.7) | 0.08 |
| Antithymocyte globulin, N (%) | 467 (56.0) | | 51 (43.6) | 416 (58.0) | 0.08 |
| *Maintenance immunosuppressive regimen* | | |  |  |  |
| Calcineurin inhibitors, N (%) |  | |  |  |  |
| Ciclosporin | 114 (13.7) | | 25 (21.4) | 89 (12.4) | 0.12 |
| Tacrolimus | 742 (89.0) | | 105 (89.7) | 637 (88.8) | 1 |
| Mycophenolate mofetil, N (%) | 742 (89.0) | | 100 (85.5) | 642 (89.5) | 0.716 |
| mTOR inhibitors, N (%) | 89 (10.7) | | 15 (12.8) | 74 (10.3) | 0.952 |
| Corticosteroids, N (%) | 834 (100) | | 117 (100) | 717 (100) |  |

SD: standard deviation; KT: kidney transplantation; IQR: interquartile range; SOT: solid organ transplantation; HIV: human immunodeficiency virus; HCV: hepatitis C virus; DSA: donor specific antibodies; CMV: cytomegalovirus; mAbs: monoclonal antibodies; mTOR: mammalian target of rapamycin.

* Significant missing data.

**Supplementary Table S2:** Description of opportunistic infections among patients converted to belatacept

|  | **Late OI**  **N=14** | **Early OI**  **N=7** |
| --- | --- | --- |
| Time in months from KT to conversion to belatacept, median (IQR) | **13.4 (3.3-33.6)** | **1.2 (0.5-4.6)** |
| Time in months between conversion to belatacept and OI, median (IQR) | **17.1 (11.2-24.8)** | **2.6 (2.3-2.7)** |
| ***Viral infections*** | 9 (64.3) | 5 (71.4) |
| Herpes zoster, N (%) | 4 (44.4) | 0 (0) |
| CMV disease, N (%) | 4 (44.4) | 1 (20) |
| BK virus nephropathy, N (%) | 1 (11.2) | 2 (40) |
| Adenovirus, N (%) | 0 (0) | 1 (20) |
| HHV8, N (%) | 0 (0) | 1 (20) |
| ***Fungal infections*** | 4 (28.6) | 1 (14.3) |
| *Pneumocystis jirovecii* pneumonia, N (%) | 4 (100) | 0 (0) |
| Cryptococcosis, N (%) | 0 (0) | 1 (100) |
| ***Bacterial infections*** | 1 (7.1) | 1 (14.3) |
| *Legionella*, N (%) | 0 (0) | 1 (100) |
| Non-tuberculous mycobacteria, N (%) | 1 (100) | 0 (0) |

IQR: interquartile range; KT: kidney transplantation; OI: opportunistic infection.
